# Measuring Generalization of Visuomotor Perturbations in Wrist Movements Using Mobile Phones

**DOI:** 10.1371/journal.pone.0020290

**Published:** 2011-05-24

**Authors:** Hugo Liberal Fernandes, Mark Vincent Albert, Konrad Paul Kording

**Affiliations:** 1 Department of Physical Medicine and Rehabilitation, Northwestern University and Rehabilitation Institute of Chicago, Chicago, Illinois, United States of America; 2 Instituto de Tecnologia Química e Biológica, Universidade Nova de Lisboa, Oerias, Portugal; 3 Department of Physiology, Northwestern University, Chicago, Illinois, United States of America; 4 Department of Applied Mathematics, Northwestern University, Chicago, Illinois, United States of America; Katholieke Universiteit Leuven, Belgium

## Abstract

Recent studies in motor control have shown that visuomotor rotations for reaching have narrow generalization functions: what we learn during movements in one direction only affects subsequent movements into close directions. Here we wanted to measure the generalization functions for wrist movement. To do so we had 7 subjects performing an experiment holding a mobile phone in their dominant hand. The mobile phone's built in acceleration sensor provided a convenient way to measure wrist movements and to run the behavioral protocol. Subjects moved a cursor on the screen by tilting the phone. Movements on the screen toward the training target were rotated and we then measured how learning of the rotation in the training direction affected subsequent movements in other directions. We find that generalization is local and similar to generalization patterns of visuomotor rotation for reaching.

## Introduction

In our lives we essentially never encounter the same situation twice. Due to changes in the environment, our own body and in our knowledge, the problems that we are solving are always different. Therefore, generalization is central to any behavior. We need to generalize what we learned in one situation and apply it to other similar situations. The topic of generalization is thus central to a large number of fields including cognitive science [1], [2], development [3], [4], and motor control [5], [6], [7], [8], [9], [10]. In fact, the issue of generalization is also the basis of most current techniques of machine learning and the basis of many algorithms for robot control [11], [12] and computer vision [13], [14]. Understanding generalization is important for many fields.

As generalization is such an important topic for neuroscience, many experiments and theories in the field of motor control have aimed at understanding generalization in a motor context. A good number of recent experimental studies have used the strategy of letting subjects learn about a perturbation for one kind of movement and subsequently testing how the learned behavior generalizes to other movements (e.g. [7], [8], [15], [16], [17]). These studies have found that certain aspects of perturbations generalize only to movements that are very similar to the training movements (local generalization) while other aspects generalize to a broad set of movements (global generalization).

To explain the results of these experiments a wide range of theories have been put forward. Some theories propose that the nervous system switches between a set of specialized controllers and that generalization happens when the same controller is used [18]. Other theories propose that the nervous system simply tunes the parameters in a general purpose neural network [19]. Additional theories propose that the nervous system maintains Bayesian estimates of the properties of the body and the world and constantly adapts these parameters [5]. What all these theories have in common is that they have been built based on reaching experiments and their predictions are being tested on such data. One exception to this general trend is a recent study that showed Bayesian algorithms can help understand how subjects generalize from the arm to the wrist and vice versa [7]. How similar reaching generalization is to other kinds of generalization is clearly a question demanding a more thorough investigation.

The vast majority of experiments in motor control in general and reaching in particular are done using virtual reality setups. Developing smaller and portable devices that allow performing movement psychophysics experiments promises to facilitate the recruitment of more subjects, the use of these devices in clinical settings and to lower the burden of running behavioral movement experiments.

Here we use a mobile phone to track wrist movements. Subjects can control the position of a cursor displayed on the screen of the phone and guide the cursor towards several targets by tilting the phone in the target's direction. We introduce a rotation to the cursor position during movements toward one target and measure how these perturbations affect movements into other directions. We find that tilt movements generalize locally and in a similar way to reaching movements.

## Methods

### Ethics Statement

The experimental protocol was approved by the Northwestern University Institutional Review Board and is in accordance with the Northwestern University Institutional Review Board's policy statement on the use of human subjects in experiments. Written informed consent was obtained from all participants. The Institutional Review Board of Northwestern University approved the study.

### Subjects

Seven healthy subjects (4 right-handed and 3 left-handed; 2 male, 5 female; aged 36.4±14.2 years) participated in the experiment. All were naive to the purpose of the experiment, and were paid according to their performance (13.0±0.8 USD).

### Task and protocol

Subjects held an android mobile phone with their dominant hand. They were seated and instructed to tilt the phone using their wrists. Their shoulder and elbow angles were not constrained. To start each trial subjects had to place a white cursor (2.1 mm diameter) in the center of the mobile phone screen (marked by a white cross) by holding the phone flat (perpendicular to gravity). After centering the cursor over the cross for 500 ms, a blue target (2.1 mm diameter) would appear at a distance of 1.83 cm in the mobile phone screen and subjects had to reach it by tilting the phone in that direction. Each reach had to be completed in a minimum time of 400 ms and a maximum time of 1200 ms, otherwise the trial would be repeated. Successful and unsuccessful reaches were indicated by a change of target's color to green and red, respectively. Different sounds were used at the end of each trial to distinguish between hitting the target, missing the target and not completing the movement within the required time interval. For each subject a learning target direction was randomly selected and the flanking generalization targets were displaced at angular distances of ±22.5**°**, ±45**°**, ±90**°** and 180**°**.

The experiment was divided into four blocks of trials: *Familiarization* (5 trials per target, 40 total), *Baseline* (10 trials per target, 80 total), *Learning* (160 trials only toward the learning direction) and *Testing* (10 trials per generalizing direction and 40 trials in the learning direction, 120 total). During the testing block the order of the target directions was pseudorandomized, with the training direction inserted every 3^rd^ trial. Subjects received endpoint feedback about the position of the cursor only in movements toward the learning direction. Except for trials in the familiarization block where the cursor was visible throughout the reaching movement, the cursor was only visible within 4 mm of the center and disappeared when the target appeared. A rotation of 30**°** was applied to the cursor position during movements toward the learning direction in the learning and testing blocks. This rotation was clockwise for right-handed subjects and counterclockwise for left-handed subjects. Subjects were paid a baseline of $5 plus $0.025 for each successful trial. This performance based reward included correct movements in the learning direction (with endpoint feedback condition) and movements in the generalization direction (without endpoint feedback). The compensation per trial was only shown during movements in the learning direction, and the total reward was only given at the end of each block.

### Apparatus

#### Phones

The experiments were performed on a series of T-mobile G1 phones, which use the AKM AK8976A accelerometer. The experiment was written in Java and developed for the android mobile phone operating system. The screens were 5.0×7.0 cm. The cursor position was centered on the screen when flat. Offset from the center was determined by a linear scaling of the accelerometer values in the horizontal and vertical axes of the screen. This is to a good approximation linearly related to the angles of tilt as the relevant angles were in the 20 degree range (see below). As the phone tilts, components of gravitational acceleration are measured along these axes. The cursor was moved from the center position 0.61 cm for every 1 m/s^2^ along that axis depending upon the amount of tilt. Reaching the targets required moving the cursor 1.83 cm from the center of the mobile phone screen - a tilt of approximately 18 degrees off the gravitational axis.

#### Optotrak

Mobile phone accelerometer readings were validated using the 3D Investigator Position Sensor (Northern Digital Inc.; 4Waterloo, Ontario, Canada). Technical specifications include an accuracy of 0.4 mm and a resolution of 0.01 mm.

### Data analysis

Data from left-handed subjects was rotated by 180**°** and angular direction was inverted so that the 0**°**, 90**°**, 180**°**, 270**°** absolute angles would correspond to the lateral, proximal, medial and distal directions, respectively, for all subjects.

The directional hand/cursor errors for each movement correspond to the tilt/cursor bias relative to the target direction at the endpoint. Timescales of learning were obtained by fitting exponential learning curves to individual subjects.

## Results

**Figure 1:**
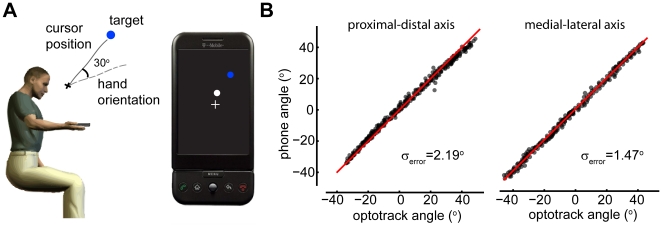
Experimental setup and validation. **A**) Subjects hold an android mobile phone with their dominant hand. They control the position of a cursor on the screen of the mobile phone by tilting it. During perturbed trials the cursor position is rotated 30° degrees relative to the true direction of tilt. **B**) Comparing the measured angle of tilt around the proximal-distal and medial-lateral axis using the optotrack versus using the mobile phone. Red line is the y = x axis.

To ask how knowledge of visuomotor perturbations generalizes from one direction of movement to other directions we conducted an experiment using an android mobile phone (see methods for detail). A cursor was presented on the screen (Fig. 1A) that indicated the tilt angle of the phone. After a block of trials to accustom subjects to the task and apparatus we introduced a perturbation during movements into the training direction. In movements toward the training direction, subjects obtained feedback at the end of every trial, which allowed them to learn about the perturbation. After learning, we interspersed trials where subjects moved into other directions without feedback to assess the amount of generalization.

We first need to verify that the tilt angles we can measure with the phones are an accurate measure of the actual tilt used by our subjects. For this, we repeatedly tilted the phone approximately 30**°** from horizontal. We measured the actual orientation of the phone in space using a 3D optical motion tracking system (see methods for details) and simultaneously recorded the tilt of the phone relative to gravity as measured by the built in accelerometers (Fig. 1B). We find that the accelerometers allow for a precise measurement of the tilt angle of the device with a standard error of approximately 2 degrees. Phones can thus be used as a precise way of running behavioral movement experiments involving tilt relative to gravity.

**Figure 2:**
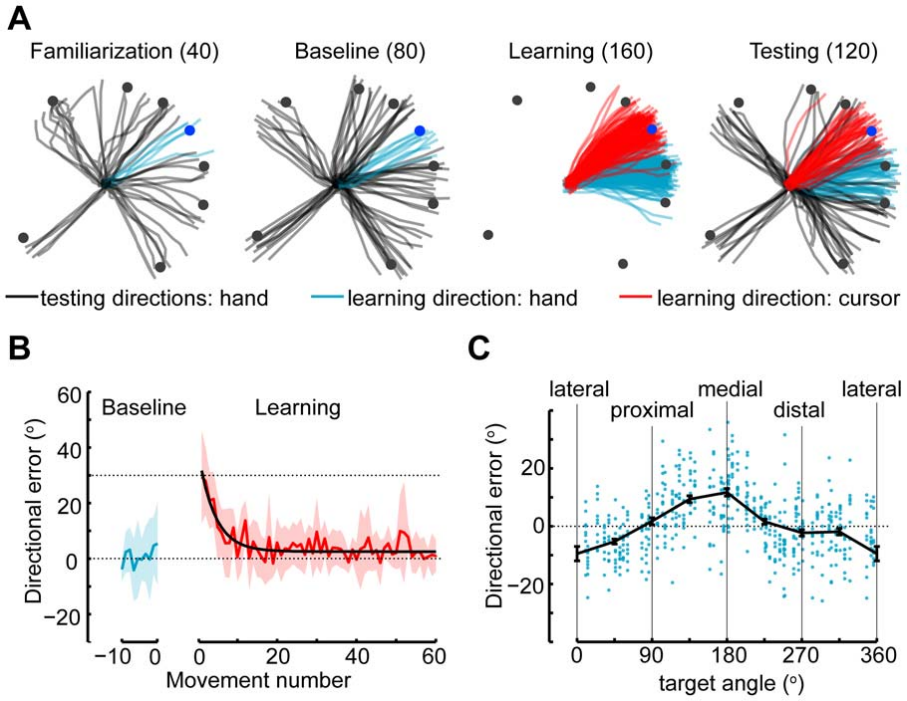
Protocol, learning and movement baseline. **A**) The four blocks of the experiment and corresponding number of trials. Lines are cursor position and hand orientation from an individual subject. **B**) Blue and red lines are average directional error of cursor (±SD) across subjects during the baseline and learning blocks. Black line is the fit of an exponential learning curve. **C**) Anisotropy of baseline movements. Average directional bias (±SEM) across subjects using 45° bins. Dots are individual trials.

Subjects were incentivized to move the cursor from a starting position toward a given target position that varied across trials. Most of the trials were toward one direction, the learning direction, which was drawn randomly for each subject. The experiment started with a familiarization block, which was followed by a baseline block and a range of learning trials (Fig. 2A). During the baseline block, subjects showed rather small variation of movement across trials toward the same direction (std = 8.0**°±**2.9**°**). Subjects can thus successfully perform target directed movements using wrist movements.

We find that subjects readily learn the visuomotor perturbation from endpoint feedback (Fig. 2B). Learning happens with a time constant of 7.9±6.8 trials (timescales from exponential fits). The timescale we observed is in line with those found in previous related papers [15], [16], [17], [20]. Visuomotor rotations of the wrist appear to be learned in a similar fashion to visuomotor rotations that affect reaching.

We found that the precision of movements during baseline (Fig. 2C) shows anisotropy across directions. Movements to targets close to the medial and lateral directions were biased toward the distal direction by approximately 10**°**. This bias might reflect biomechanical effects or biases in perception. The bias that affects subjects appears to be relatively large in comparison to the typical standard deviation of movements.

**Figure 3:**
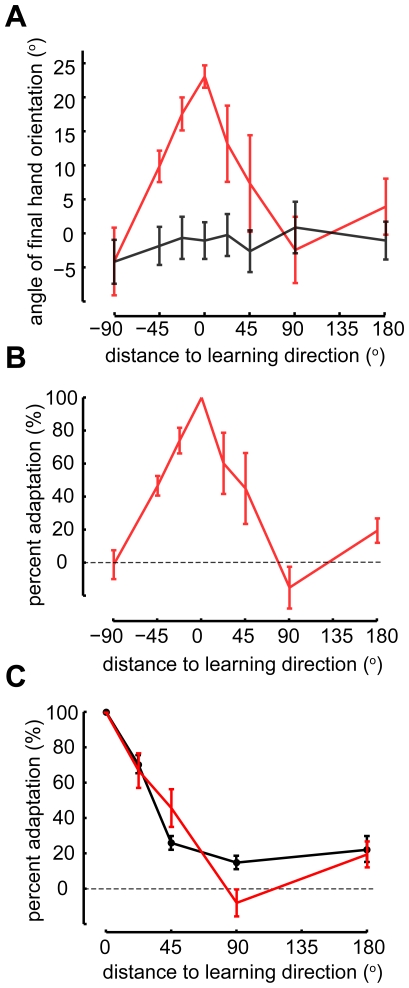
Generalization patterns. **A**) Baseline (black) and generalization (red) of the rotation across multiple directions (±SEM). **B**) Percent adaptation (±SEM) in the generalizing directions relative to the learning direction. **C**) Percent adaptation (±SEM) for a visuomotor rotation in a center-out reaching task [16] (black) overlapped with data from Fig. 3b (red). Data from targets at the same absolute distance from the learning direction were combined.

The main question we are asking in this study is how subjects generalize the learned behavior. We find that learned visuomotor perturbations generalize locally (Fig. 3A,B). There was no influence of learning on movements into directions 90**°** away from the training direction (p>0.48, one sided t-test). Comparing to the results from an analogous study that used reaching [16] (Fig. 3C) there appears to be differences at 45**°** and 90**°** but they are not statistically significant (p = 0.11 for 45**°** and p = 0.06 for 90**°**, t-test with Bonferroni correction). Moreover, there is a weak non-zero generalization at 180**°** (p<0.05, one sided t-test) both in our data and in previous reaching data. Generalization of visuomotor rotations for wrist movements appears to be local and qualitatively similar to generalization of reaching movements.

## Discussion

Here we measured the generalization curves for visuomotor rotations applied to wrist movements. We found that subjects readily learn such perturbations and generalize locally, in a similar way to previously measured generalization in reach adaptation studies. Furthermore, we have also established the use of mobile phones to run movement experiments in motor control.

We have found generalization to be local for wrist movements during tilt adaptation, and it is interesting to speculate why it is so. One way of interpreting these results is in terms of tuning properties in the nervous system. It may be that both for reaches and for wrist movements, neurons have narrow tuning to direction of movement, and as learning happens in these populations it generalizes locally [8], [15], [16]. An alternative and complementary interpretation may be that generalization is local because relevant changing properties of the body and the environment differ if movements are dissimilar [5]. Independently of the interpretation, we observe local adaptation which matches results from previous studies.

The adaptation at 180 degrees can also be observed in reaching experiments using visuomotor rotation [16] or force field adaptation [21]. When subjects are returning to the start position they inevitably move in a 180 degree direction relative to the learning direction. Subjects may be adapting to the perturbation during these movements. Alternatively, one might hypothesize that this adaptation can be understood from the tuning properties of neurons in the nervous system. There is a small number of corticomotor (16% in [22]) and cerebellar (∼15% in [23]) neurons which exhibit bimodal tuning properties. If adaptation relies on both unimodal, traditionally cosine-tuned cells, and these bimodal cells then we might expect a small visible bimodal adaptation. Behaviorally, the reason for this non-local adaptation may be the strong association between opposing directions of movement. This non-local 180 degree adaptation is indicated by our task and previous reaching studies, and can be interpreted in terms of both known neurophysiology and behavioral context.

We found clear anisotropy during movement with biases generally being into the distal direction. The existence of biases should not be overly surprising; there is a clear anisotropy in the biomechanics of wrist movement. There is also the potential for biases coming from the cardinal axes of the phone and related perception. During everyday life people often move the phone from their ears into a horizontal position and these natural statistics [24], [25] might affect targeted movements. While the origin of these effects is interesting we focused here on the generalization curves.

While generalization is usually probed using reaching experiments our results show that local generalization is also a feature of the motor system outside of reaching. Local generalization affects wrist movements and we would predict that it would equally affect posture and movements of the feet and head.

Our application adds to the growing literature of using mobile phones in medical contexts. For example, phones have been used to measure the type of physical activity [26], monitor people with chronic medical conditions [27], and detect falls [28]. Cheaper and more versatile ways of collecting data like in this study can make the recording and application of movement data much more ubiquitous. From clinical populations to populations in hard to reach areas of the world, mobile phones provide a useful tool for studying and using movement data.

## References

[1] Shepard RN (1987). Toward a Universal Law of Generalization for Psychological Science.. Science.

[2] Chater N, Vitanyi PMB (2003). The generalized universal law of generalization.. Journal of Mathematical Psychology.

[3] Quinn PC, Tanaka JW (2007). Early development of perceptual expertise: Within-basic-level categorization experience facilitates the formation of subordinate-level category representations in 6- to 7-month-old infants.. Memory & Cognition.

[4] Smith LB (1979). Perceptual Development and Category Generalization.. Child Development.

[5] Berniker M, Kording K (2008). Estimating the sources of motor errors for adaptation and generalization.. Nature neuroscience.

[6] Goodbody S, Wolpert D (1998). Temporal and amplitude generalization in motor learning.. Journal of Neurophysiology.

[7] Krakauer J, Mazzoni P, Ghazizadeh A, Ravindran R, Shadmehr R (2006). Generalization of motor learning depends on the history of prior action.. PLoS Biol.

[8] Thoroughman K, Shadmehr R (2000). Learning of action through adaptive combination of motor primitives.. Nature.

[9] Shadmehr R, Moussavi ZMK (2000). Spatial generalization from learning dynamics of reaching movements.. Journal of Neuroscience.

[10] Donchin O, Francis JT, Shadmehr R (2003). Quantifying generalization from trial-by-trial behavior of adaptive systems that learn with basis functions: Theory and experiments in human motor control.. Journal of Neuroscience.

[11] Calinon S, Guenter F, Billard A (2007). On learning, representing, and generalizing a task in a humanoid robot.. Ieee Transactions on Systems Man and Cybernetics Part B-Cybernetics.

[12] Sutton RS (1996). Generalization in reinforcement learning: Successful examples using sparse coarse coding.. Advances in Neural Information Processing Systems.

[13] Poggio T, Bizzi E (2004). Generalization in vision and motor control.. Nature.

[14] Wright J, Yang AY, Ganesh A, Sastry SS, Ma Y (2009). Robust Face Recognition via Sparse Representation.. Ieee Transactions on Pattern Analysis and Machine Intelligence.

[15] Paz R, Boraud T, Natan C, Bergman H, Vaadia E (2003). Preparatory activity in motor cortex reflects learning of local visuomotor skills.. Nature neuroscience.

[16] Krakauer J, Pine Z, Ghilardi M, Ghez C (2000). Learning of visuomotor transformations for vectorial planning of reaching trajectories.. Journal of Neuroscience.

[17] Mattar A, Ostry D (2007). Modifiability of generalization in dynamics learning.. Journal of neurophysiology.

[18] Haruno M, Wolpert D, Kawato M (2001). Mosaic model for sensorimotor learning and control.. Neural Computation.

[19] Spivey J (2007).

[20] Hinder MR, Tresilian JR, Riek S, Carson RG (2008). The contribution of visual feedback to visuomotor adaptation: How much and when?. Brain research.

[21] Donchin O, Francis JT, Shadmehr R (2003). Quantifying generalization from trial-by-trial behavior of adaptive systems that learn with basis functions: theory and experiments in human motor control.. Journal of Neuroscience.

[22] Amirikian B, Georgopulos AP (2000). Directional tuning profiles of motor cortical cells.. Neuroscience Research.

[23] Coltz J, Johnson M, Ebner T (1999). Cerebellar Purkinje cell simple spike discharge encodes movement velocity in primates during visuomotor arm tracking.. The Journal of neuroscience.

[24] Slijper H, Richter J, Over E, Smeets J, Frens M (2009). Statistics predict kinematics of hand movements during everyday activity.. Journal of Motor Behavior.

[25] Howard I, Ingram J, Kording K, Wolpert D (2009). Statistics of Natural Movements Are Reflected in Motor Errors.. Journal of neurophysiology.

[26] Brezmes T, Gorricho JL, Cotrina J (2009). Activity Recognition from Accelerometer Data on a Mobile Phone.. Distributed Computing, Artificial Intelligence, Bioinformatics, Soft Computing, and Ambient Assisted Living, Pt Ii, Proceedings.

[27] Mathews AG, Butler R (2005). A vision for the use of Proactive mobile computing tools to empower people with chronic conditions.. 18th IEEE Symposium on Computer-Based Medical Systems, Proceedings.

[28] Rohs M, Gfeller B (2004). Using camera-equipped mobile phones for interacting with real-world objects.. Advances in Pervasive Computing.

